# Hematopoietic Stem-Cell Transplantation in the Resource-Limited Setting: Establishing the First Bone Marrow Transplantation Unit in Bangladesh

**DOI:** 10.1200/JGO.2016.006460

**Published:** 2016-12-21

**Authors:** Albert C. Yeh, Mohiuddin A. Khan, Jason Harlow, Akhil R. Biswas, Mafruha Akter, Jannatul Ferdous, Tasneem Ara, Manirul Islam, Martin Caron, Anne-Marie Barron, Jenna Moran, Mark Brezina, Humayra Nazneen, Md Kamruzzaman, Anup Saha, Ariela Marshall, Salma Afrose, Christopher Stowell, Frederic Preffer, David Bangsberg, Annekathryn Goodman, Eyal Attar, Steven McAfee, Thomas R. Spitzer, Bimalangshu R. Dey

**Affiliations:** **Albert C. Yeh**, **Christopher Stowell**, **Frederic Preffer**, **Eyal Attar**, **Steven McAfee**, **Thomas R. Spitzer**, and **Bimalangshu R. Dey**, Massachusetts General Hospital; **Jason Harlow** and **David Bangsberg**, Massachusetts General Hospital Center for Global Health; **Martin Caron**, **Jenna Moran**, **Mark Brezina**, **Eyal Attar**, **Steven McAfee**, **Thomas R. Spitzer**, and **Bimalangshu R. Dey**, Massachusetts General Hospital Bone Marrow Transplant Program; **Anne-Marie Barron**, Simmons College School of Nursing and Health Science; **Annekathryn Goodman**, **Eyal Attar**, **Steven McAfee**, **Thomas R. Spitzer**, and **Bimalangshu R. Dey**, Massachusetts General Hospital Cancer Center, Boston, MA; **Ariela Marshall**, Mayo Clinic, Rochester, MN; and **Mohiuddin A. Khan**, **Akhil R. Biswas**, **Mafruha Akter**, **Jannatul Ferdous**, **Tasneem Ara**, **Manirul Islam**, **Humayra Nazneen**, **Md Kamruzzaman**, **Anup Saha**, and **Salma Afrose**, Dhaka Medical College and Hospital, Dhaka, Bangladesh.

## Abstract

**Purpose:**

Treatment of malignant and nonmalignant hematologic diseases with hematopoietic stem-cell transplantation (HSCT) was first described almost 60 years ago, and its use has expanded significantly over the last 20 years. Whereas HSCT has become the standard of care for many patients in developed countries, the significant economic investment, infrastructure, and health care provider training that are required to provide such a service have prohibited it from being widely adopted, particularly in developing countries.

**Methods:**

Over the past two decades, however, efforts to bring HSCT to the developing world have increased, and several institutions have described their efforts to establish such a program. We aim to provide an overview of the current challenges and applications of HSCT in developing countries as well as to describe our experience in developing an HSCT program at Dhaka Medical College and Hospital in Bangladesh via a partnership with health care providers at Massachusetts General Hospital.

**Results and Conclusion:**

We discuss key steps of the program, including the formation of a collaborative partnership, infrastructure development, human resource capacity building, and financial considerations.

## INTRODUCTION

Hematopoietic stem-cell transplantation (HSCT) has the potential to treat many congenital and acquired diseases of the hematopoietic system, and its use has expanded significantly over the last 20 years as a result of the availability of new technologies, including the use of peripheral and cord blood as a source of stem cells, development of a worldwide donor registry, and use of low-intensity conditioning regimens in older patients.^1-5^ There is evidence for use of HSCT in malignant conditions, such as non-Hodgkin lymphoma, acute myeloid leukemia, myelodysplastic syndrome, acute lymphoid leukemia, chronic myeloid leukemia, and multiple myeloma, and for several nonmalignant hematologic disorders, including sickle-cell anemia, thalassemia, and inherited immunodeficiencies.^6-10^ Although HSCT is used in many locations worldwide, the transplantation process is complex and costly. During the first 100 days alone, the median cost is estimated to be more than $200,000 USD for allogeneic transplantation and $100,000 for autologous transplantation.^11^ In addition to financial support, successful HSCT requires specialized infrastructure and extensive health care provider training. Gratwohl et al^12^ conducted a global assessment of HSCT procedures and found that rates of HSCT use were highly associated with countries with higher gross national income per capita, governmental health care expenditures, and human development index. For these reasons, HSCT is more common in affluent countries. Nevertheless, interest in developing HSCT programs for resource-limited settings has steadily increased, and several countries have described successful programs for both autologous and allogeneic transplantation (Table 1).^13-17^ The potential ability for HSCT to cure certain chronic, debilitating diseases, such as transfusion-dependent thalassemia in children,^17^ may economically justify its use relative to other treatments available when accounting for long-term costs.

**Table 1:**
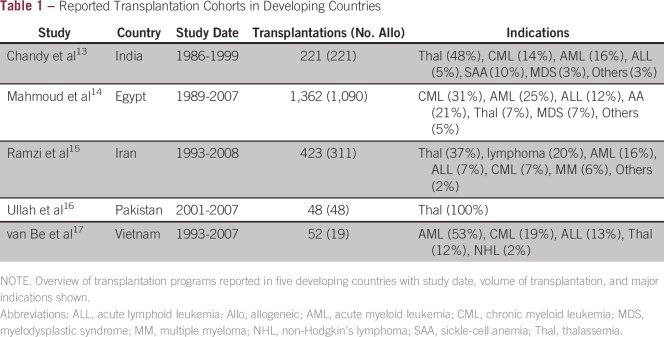
Reported Transplantation Cohorts in Developing Countries

## HEALTH CARE IN BANGLADESH

Bangladesh—one of the most populous countries in the world—has been plagued by pervasive poverty, income inequality, and fragmented political parties since its independence in 1971; however, the Bangladeshi government, with the assistance of international organizations, has vigorously pursued the improvement of health care outcomes over the past several decades. Recent improvements in economic development have resulted in improved health metrics, such as those for infant, child, and maternal mortality.^18^

**Fig 1:**
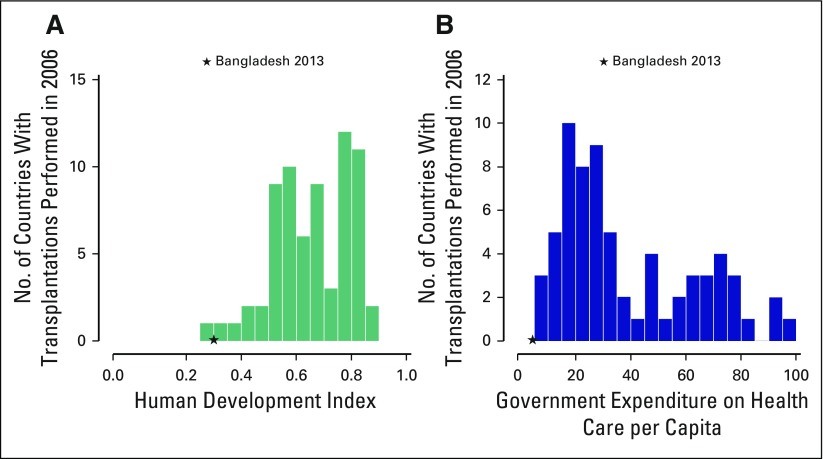
Human development index and government expenditure in countries with transplantation programs. (A) Countries with capacity for hematopoietic stem-cell transplantation (HSCT) versus human development index. Units for the human development index are squared. Data were derived from the United Nations Development Program.^21^ (B) Countries with capacity for HSCT versus government expenditure on health care per capita. Units for health care per capital are provided as square roots. Data were derived from The World Bank.^22^ The star marks the position of Bangladesh in 2013 for these two parameters. Countries with transplantation programs were derived from Gratwohl et al.^12^

Despite its recent advancements, however, Bangladesh has little infrastructure to support the complex, quaternary level of care that is required for a successful HSCT program. Bangladesh has been identified by the WHO as one of a handful of countries with a severe shortage of human resources for health, with approximately three physicians and one nurse per 10,000 people.^19,20^ Using metrics set out by Gratwohl et al to predict a country’s capacity for HSCT, Bangladesh falls on the lowest end of the spectrum with respect to both government expenditure on health per capita and the human development index compared with other countries that have existing programs (Figs 1A and 1B).

## DHAKA MEDICAL COLLEGE AND HOSPITAL

The Dhaka Medical College and Hospital (DMCH) was established in 1946 under British colonial rule and is located in the heart of Dhaka City. DMCH is a public hospital and its operations are funded entirely by the government of Bangladesh. There are approximately 250 attending physicians and 600 nurses to staff both the inpatient and outpatient services. Similar to most public hospitals in this region, the volume of patients is large. DMCH sees 5,000 to 6,000 patients per day in its outpatient services, and manages 2,300 inpatient beds located in two buildings—DMCH-1, with 1,800 beds, and DMCH-2, which was built in 2013, with 500 beds (Data Supplement). DMCH routinely runs at 20% to 30% over its inpatient capacity, and many patients are treated outside the wards in open hallways.

## INTEREST IN BONE MARROW TRANSPLANTATION AT DMCH

In 2012, the Ministry of Health and Family Welfare (MOHFW) and political leadership of Bangladesh became interested in establishing an HSCT program at DMCH. On the basis of the reputation of Massachusetts General Hospital (MGH) for its global health initiatives, MOHFW approached transplantation physicians at the MGH Cancer Center to develop a national transplantation program. The intention of the program was to provide treatment of nonmalignant hematologic disorders that are curable by HSCT, both in Bangladesh and for the region. The Bangladesh government provided funding for the endeavor. The initial goal was to build a comprehensive program and center of excellence for hematologic malignancies. We discuss four key aspects of the program, including: (1) formalizing of a collaborative partnership, (2) infrastructure development, (3) human resource capacity building and implementation of the concept of clinical teamwork, and (4) financial considerations. We also provide a brief summary of the initial outcomes of our first 21 transplantations.

## FORMALIZING A COLLABORATIVE PARTNERSHIP

To codify the scope and scale of the collaboration, a memorandum of understanding (MOU) was signed between MOHFW and MGH. With the specific aim of improving oncology services for the people of Bangladesh, the MOU summarized the agreement between the parties to collaborate in (1) the exchange of faculty and staff, (2) the exchange of students (undergraduate, medical, and postgraduate), (3) joint capacity building in specific clinical specialties (eg, nursing), and (4) joint monitoring and evaluation of shared activities. Specific terms of collaboration for each activity were outlined to provide a detailed framework for the scale and scope of the collaboration. The MOU also included standard institutional protections, including (1) terms of confidentiality, (2) validity, (3) process and terms of termination, (4) governing laws and language for resolution of disputes, and (5) standard language governing incidents of force majeure.

As the parties recognized that the scope of activities within the partnership could evolve over time, MOHFW and MGH also agreed to attach and periodically revise them to adjust specific scopes of work to govern the annual work plans for each partner. These scopes of work documents were attached as amendments to the MOU.

## INFRASTRUCTURE DEVELOPMENT

A strong indication of support from the highest levels of the government of Bangladesh was the MOHFW commitment to building a state-of-the-art facility. Occupying the top floor of DMCH-2, the new HSCT unit was based on physical layout and mechanical designs that were used in the transplantation unit at MGH (Data Supplement). The DMCH-2 HSCT unit is approximately 7,000 square feet and includes five private inpatient rooms, an apheresis area, a stem-cell processing laboratory, hematopathology and general hematology laboratories, and shared inpatient rooms for patients with leukemia who have not yet undergone HSCT. The company that oversaw the construction of multiple clinical buildings at MGH supported the planning and construction process in Dhaka, including an in-person consulting visit to Bangladesh by the chief engineering officer. Aside from the physical space itself, transplantation centers in the developing world face the unique challenge of having to provide high-level care on a strict budget, which may result in poorer outcomes compared with transplantations performed in developed countries. Critical components of a functioning transplantation program include basic infrastructure and sanitation as well as reliable protocols for transfusion medicine, pharmacy, and diagnostics. After close examination of the work in progress, the local construction and engineering teams received critical recommendations that were incorporated with the objective of enhancing the care of sick patients who undergo transplantation, including reducing the risk of infection-related complications. Some of the feedback and recommendations from external engineering reviews are described below.

#### Air and Water Handling Systems

Transplantation centers in resource-limited settings must effectively address basic sanitation needs, including appropriate air handling and water units. Chandy et al^13^ provided valuable insights from their experience in establishing an allogeneic HSCT program in India and emphasized that, in this region of the world, the poorer quality of the ambient environment and the higher level of antimicrobial resistance make it particularly crucial to have a transplantation unit with strict environmental control. Other programs that have been established in developing countries describe similar challenges.^14,16^ Feedback by an experienced consultant team from India made several specific recommendations regarding the air handling unit and water system at DMCH. Emphasis was placed on establishing regular logs and monitoring of air particle and water colony counts, as rates of fungal infections and coliform counts in the water supply are generally higher in this region. Another strong recommendation was to have reliable standby systems in place, as the quality, technical knowledge, and speed of service may hinder the ability to recover from potential down times.

#### Apheresis, Cell Processing, and Laboratory Facilities

Apheresis capacity, cell processing, blood banking, and diagnostics are fundamental components of a transplantation center that requires standard operating protocols as well as particular equipment and trained personnel. Diagnostic capabilities at DMCH include two flow cytometers and the ability to perform basic cytology. More advanced diagnostics, including cytogenetics, fluorescent in situ hybridization, and PCR, are sent to neighboring private hospitals or to India. Whereas the regional consultant team noted that these facilities are excellent for a unit that is just starting, several specific recommendations were also made, including using in-line uninterruptible power supply systems for critical operations, such as apheresis machines and controlled rate freezers, having systems in place for quality control temperature maintenance of liquid nitrogen storage tanks and −80°C freezers, and taking part in an external quality assurance program for CD34 counts.

## HUMAN RESOURCE CAPACITY BUILDING AND CLINICAL TEAMWORK

**Fig 2:**
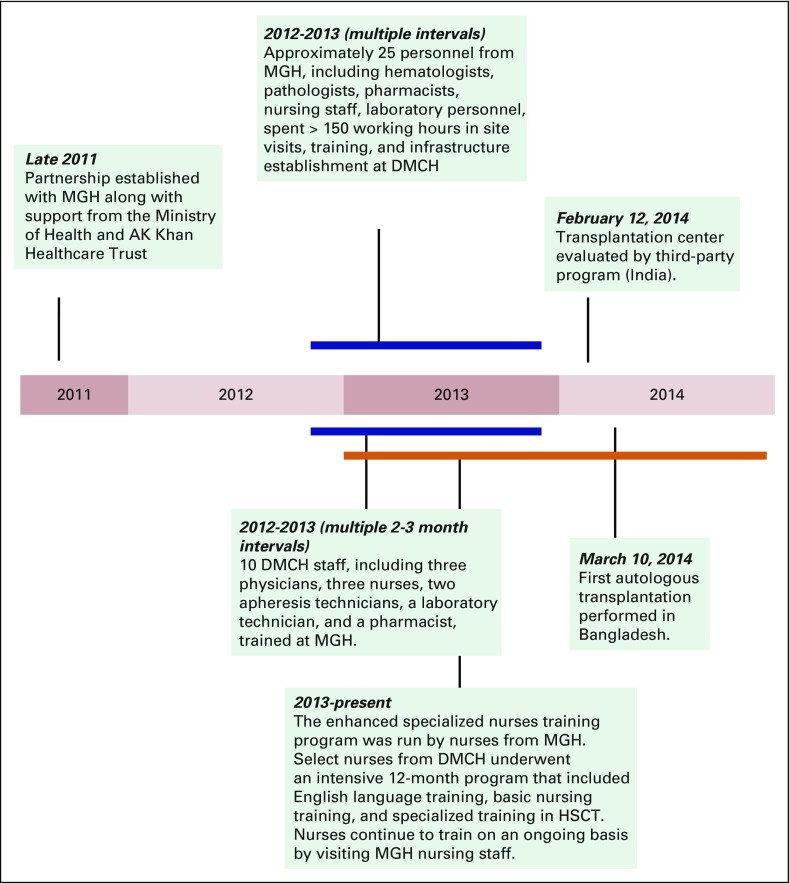
Development timeline. A highlight of key events for the Dhaka Medical College and Hospital (DMCH) transplantation program is shown, from conception in 2011 until the first autologous transplantation on March 10, 2014. HSCT, hematopoietic stem-cell transplantation; MGH, Massachusetts General Hospital.

Integration of specialized nurses, physicians, and support staff is essential for the care of patients who undergo HSCT and represented a significant undertaking. Education and training of key personnel required an ongoing bidirectional exchange of MGH and DMCH staff as well as the establishment and dissemination of training curricula. In an effort to make this training as relevant to the local context as possible, more than 40 physicians, nurses, laboratory technologists, pharmacists, and administrators from MGH spent more than 150 weeks in Dhaka training and advising the HSCT program. Training programs began in 2012 and were completed by 2014 (Fig 2). Detailed training manuals and practical examinations were used for quality control and to assess trainee progress (available upon request).

### Nursing Staff

There is no specialty training program for nurses in either hematology or bone marrow transplantation at DMCH, and a key challenge in establishing the HSCT program was the need to improve the level of nursing care, including enhancing the clinical knowledge base as well as integrating nurses into daily rounds. In experienced transplantation centers, nurses typically assume a large degree of responsibility for identifying and managing complications during the peritransplant period and play a critical role on the health care team. In contrast, the level of training and the degree of professional independence afforded nurses in resource-limited settings impedes the performance of the entire care team.

In Bangladesh, the nursing community has been historically undervalued: nurses have a much more limited scope on medical rounds and are not empowered to serve as effective patient advocates. Given the critical role nurses have in the day-to-day management of patients who undergo transplantation, a central goal for the collaboration between MGH and the team at DMCH was to introduce the idea of team-based rounds during which all medical staff, including nurses, share equal responsibility in raising patient-related issues and in presenting clinical data and professional opinions. To elevate the clinical competencies and professional status of the nurses in the DMCH HSCT unit, a comprehensive curriculum for the care of patients who undergo HSCT was developed by nurses and nurse practitioners at MGH and the Simmons College School of Nursing and Health Sciences. Training Bangladeshi nurses in HSCT management techniques was an extensive undertaking that is described in detail in a companion report by Barron et al^23^ on the enhanced specialized nurses training program.

### Transfusion Services

A needs assessment, performed in Dhaka by a transfusion medicine physician, an apheresis nurse, and a stem-cell laboratory technologist, formed the basis for a staged plan to put the necessary infrastructure for transfusion services in place. This infrastructure included equipping a stem-cell processing laboratory, purchasing an additional apheresis instrument for both stem-cell and platelet collections, installing a dedicated blood irradiator, developing the ability to leukoreduce cellular blood components, expanding platelet collections, developing systems for transport and tracking of blood components, and staff training. The first step in training was to bring key staff from Dhaka to MGH to train in the collection of peripheral blood stem cells; stem-cell processing, including freezing, storing, and preparing for transplantation; and processing of blood components for transfusion to patients undergoing bone marrow transplantation. The same transfusion service team that performed the needs assessment later returned to Dhaka to verify that systems were in place to support these patients, and was present at the time of the first several collections.

Over the course of this venture, DMCH laboratory technologists were trained under the same platform as a new MGH employee. This included aspects of quality control, quality assurance, and techniques for cellular therapy processing and infusion. This training took place at MGH for approximately 3 months and then at DMCH for approximately 1 month. Technologists shadowed MGH technologists and were eventually tested and qualified to perform all processes independently. Once the laboratory was built in Dhaka, equipment was qualified before use and essential equipment used by MGH was duplicated for familiarity. Standard operating procedures were written to reflect the processes that the DMCH laboratory would use and was critical to facilitating the initial transition. Before the laboratory opened, a full review was performed by the chief technologist of the bone marrow processing laboratory at MGH. MGH bone marrow processing staff were on site during the first four collections to assist DMCH technologists with processing.

### Hematology Diagnostic Laboratory

The hematology laboratory was enhanced with basic flow cytometry capabilities, and DMCH staff were trained in basic diagnostics and analysis of CD34 counts for stem-cell collection. Two technologists and hematologists received training from two MGH flow cytometry senior technicians in Dhaka, who made multiple trips and were present during the first few stem-cell collections. One hematologist spent 2 months at the MGH flow cytometry laboratory and learned directly from the technicians as well as from the senior pathologist.

### Pharmacy

The critical role played by specialized pharmacy personnel in the management and delivery of transplantation regimens was also addressed through personnel exchange and ongoing communication between MGH and DMCH. The head pharmacist from DMCH spent approximately 2 months at MGH to gain the practical knowledge and compounding skills that were necessary to administer toxic chemotherapy. Personnel from MGH also spent 4 to 6 weeks at DMCH to educate the physicians, nurses, and pharmacy staff about standard HSCT regimens, including the country’s first exposure to busulfan/cyclophosphamide and BEAM (carmustine, etoposide, cytarabine, and melphalan) protocols.

### Physicians

The hematology department at DMCH consists of six faculty physicians and 20 physician trainees. With little previous exposure to transplantation medicine, three hematologists spent 3 months at MGH as clinical observers of various aspects of HSCT, including diagnostics, peripheral blood stem-cell collection, stem-cell processing and preservation, and blood transfusion medicine. Because the care of patients who undergo HSCT often involves the expertise of other disciplines, special arrangements were made with the intensive care unit (ICU) and pulmonary and cardiology services at DMCH to provide consultants to regularly visit patients undergoing HSCT who had specific medical concerns at the time of admission. One senior physician from Dhaka received training at the Transplantation Infectious Disease Unit at MGH to support the diagnostic work-up and management of infection-related problems. Similarly, the radiology department provided specialized services to patients undergoing HSCT, including computed tomography scans, and the ICU team provided on-site support for patients undergoing HSCT who had cardiorespiratory distress, thereby avoiding patient transfers to the general ICU and significantly diminishing the risk of infection.

## FINANCIAL CONSIDERATIONS

Similar to other public sector services in Bangladesh, the long-term viability and maintenance of public health care programs are dependent on government support. An estimation of the initial costs to establish the transplantation unit, including floor space, materials and supplies, and personnel is provided (Data Supplement). One of the major motivating factors that drove this support was the potential for the HSCT program to not only offer a more effective way to treat certain hematologic malignancies but also to provide a more cost-effective approach to manage specific chronic conditions, particularly thalassemia, which is the most common genetic disorder in this region.

In Bangladesh, approximately 3% to 4% of the population are carriers of β-thalassemia and 4% to 6% are carries of hemoglobin E.^24^ More than 7,000 children are diagnosed with thalassemia major each year in Bangladesh, but they typically do not survive for more than 5 years without chronic blood transfusions.^25^ A child who is afflicted with thalassemia major will likely need frequent transfusions before age 1 year and iron chelation therapy later in childhood. It is estimated that the cost per year of treating thalassemia in developing countries, such as Sri Lanka, Thailand, and Bangladesh, ranges from approximately $1,000 to $2,500 per year, including costs of transfusions, chelating agents, and hospital visits.^26,27^ Excluding societal costs that are associated with loss of productivity, the ability to cure these patients via HSCT offers the potential to alleviate long-term maintenance costs, which can surpass the upfront cost associated with transplantation within 10 to 20 years.

Whereas the initial cost of an allogeneic transplantation is approximately $200,000 in developed countries,^28^ similar protocols in a developing country generally cost almost an order of magnitude less, ranging from approximately $10,000 to $25,000.^13-17^ Even at these lower costs, transplantation remains prohibitively expensive for most of the population in these countries. For example, whereas a recent report suggested that the average upfront allogeneic transplantation in India costs approximately $17,000, only approximately 5% of the population is able to afford a transplantation.^13^ Third-party funding mechanisms, through governmental or nongovernmental organizations, are required to increase access. In Egypt, which now has eight transplantation centers, patients rely on fully sponsored insurance from the Ministry of Health and the country performs around 170 transplantations a year.^14^ In Pakistan, the Armed Forces Bone Marrow Transplant Center provides 35 to 40 transplantations per year and completely subsidizes the costs for military personnel and their families.^16^ At DMCH, the MOHFW has supported 21 autologous transplantations to date, with an initial transplant cost of approximately 700,000 TK (approximately $9,000 USD). More complex and resource consuming, allogeneic transplantation is projected to cost between $15,000 and $18,000.

## SUMMARY OF INITIAL OUTCOMES

As of May 2016, 21 patients (age range, 18 to 58 years) had undergone autologous transplantations at DMCH. We have treated 11 patients with myeloma, four with diffuse large B-cell lymphoma, four with Hodgkin lymphoma, one with acute myelogenous leukemia, and one with peripheral T-cell lymphoma. Conditioning regimens used include melphalan,^11^ BEAM,^9^ and busulfan/cyclophosphamide.^1^ Engraftment, as defined by sustained platelet count greater than 20 ×10^9^/L and neutrophil count greater than 0.5 × 10^9^/L, occurred in all patients (range 9 to 16). There were 10 documented infections, including seven cases of bacteremia, two *Clostridium difficile* infections, and one case of pneumonia. There have been no transplantation-related mortalities (TRMs) to date. Five patients have experienced relapse (ranging from day 213 to day 598), and the longest disease-free survivor is now 639 days out from transplantation.

## DISCUSSION

Our experience working with DMCH in establishing a global partnership, developing infrastructure, and building human resource capacity highlights not only several challenging hurdles that may be faced when establishing a quaternary-level resource in the developing world, but the importance of regional considerations as well. Some of these factors, such as the status and role of nurses, require investment in resources that are beyond the scope of the immediate program. We also acknowledge that the maintenance of such a program, once developed, involves addressing future questions and challenges that may arise. For example, the economic justification of transplantation for thalassemia is a decision that will ultimately be made by the government as it decides how to allocate scarce funding resources. From our experience, the most important factors that contributed to the successful establishment of Bangladesh’s first transplantation program include the unwavering commitment from the MOHFW to establish a robust collaboration with an experienced transplantation center that is able to provide the expertise, training, and overall guidance to a complex and multidimensional program. Although our experience is limited given the small number of patients who have undergone autologous HSCT, we believe that the collaborative efforts between DMCH and MGH can help serve as a model for future partnerships in clinical hematology oncology.

A few features distinguish the program’s development over a relatively short timeframe. First, significant and routine guidance from experienced practitioners at MGH, especially during initial stages, provided critical expertise to ensure adherence to protocols and to troubleshoot complications in real time. In addition, the ongoing dissemination and maintenance of knowledge and expertise was facilitated by semiannual professional development visits of several physicians from DMCH to MGH for continued training in transplantation protocols, blood banking, diagnostics, and flow cytometry. MGH also continues to send nursing staff as part of its ongoing educational program to elevate the level of nursing care at DMCH.

With respect to outcomes, whereas we have only given transplantations to a handful of patients, we have also—reassuringly—not experienced any TRMs. Although multiple variables likely contribute to TRMs, we believe that it could be a novel outcome measure to evaluate both the collaboration and training elements of the program. TRM is intimately tied to strict adherence to indications and contraindications of transplantation to deliver the right treatment to the right patient, active patient monitoring, warm handoffs of clinical responsibilities between clinicians, unerring fidelity to infection control protocols, and a patient-centered, team-based approach to clinical care. Another important area that will also help to improve outcomes is patient counseling, which is currently performed by the physician during the initial and follow-up visits, with nursing staff involved during inpatient care. Although we do not currently have ancillary staff services to support this endeavor in the outpatient setting, we hope to improve upon this effort, in particular, by adding this component as part of the nursing training program in the future.

We are preparing for the country’s first allogeneic transplantation in 2017. As Bangladesh does not have an existing bone marrow registry, the initial focus will be on transplantation with matched sibling donors. In the meantime, efforts to expand the donor base will involve increased outreach to raise awareness and acceptance of HSCT as well as potential collaborative efforts with neighboring countries that have existing donor registries. There will be additional infrastructure needs, including developing outpatient rooms for bone marrow harvest; adding special laboratories to run levels of tacrolimus, cyclosporine, and rapamycin; providing physician and nurses with additional training on management and treatment of graft-versus-host disease; collaborating with special centers, including Apollo Hospital systems and the International Center for Diarrheal Disease Research, to run specialized studies, such as PCR, for various viral pathogens; and upgrading the pharmacy inventory for specialized antiviral, antifungal, and other antibiotics.

It is difficult to compare outcomes across transplantation centers in developing countries because of the limited sample size and heterogeneity of indications. As a result of a variety of challenges, including socioeconomic factors and higher infection risks, resource-limited countries face additional hurdles and have somewhat higher complication rates compared with their developed peers. For example, Mahmoud et al^14^ reported a significant follow-up dropout rate of 20% in the Egyptian experience, with lapses in compliance during post-transplantation hygienic care, despite being provided free of cost. Only a handful of other developing countries have been able to establish a sustainable HSCT program. Of these, the countries that are most similar to Bangladesh with respect to region, human development index, and government medical care expenditure per capital include Pakistan, Vietnam, and India. Centers in these countries have performed allogeneic transplantations for a variety of diseases, the majority for which are thalassemias.^13,16,17^ There are clearly unmet medical needs that can be addressed by transplantation, and future partnerships that are led by experienced transplantation centers will be critical for the efficient establishment and expansion of HSCT to the developing world.
